# *In vitro* and *in silico* investigations of Propolis-derived phytochemicals as potential inhibitors of *Plasmodium falciparum*

**DOI:** 10.14202/vetworld.2025.1644-1659

**Published:** 2025-06-19

**Authors:** Dhrubo Ahmed Khan, Md. Nazmul Hasan, Rachasak Boonhok, Suthinee Sungkanu, Yutatirat Singhaboot, Afsana Amin Shorna, Anamul Hasan, Kesinee Chotivanich, Polrat Wilairatana, Abolghasem Siyadatpanah, Roghayeh Norouzi, Imran Sama-ae, Watcharapong Mitsuwan, Alok K. Paul, Maria de Lourdes Pereira, Shanmuga S. Sundar, Tooba Mahboob, Christophe Wiart, Ryan V. Labana, Siriphorn Chimplee, Veeranoot Nissapatorn

**Affiliations:** 1 Laboratory of Pharmaceutical Biotechnology and Bioinformatics, Department of Genetic Engineering and Biotechnology, Jashore University of Science and Technology, Jashore, Bangladesh; 2 School of Allied Health Sciences, Research Excellence Center for Innovation and Health Products (RECIHP) and World Union for Herbal Drug Discovery (WUHeDD), Walailak University, Nakhon Si Thammarat, Thailand; 3 Department of Clinical Tropical Medicine, Faculty of Tropical Medicine, Mahidol University, Bangkok, Thailand; 4 Department of Biotechnology and Genetic Engineering, University of Development Alternative, Dhaka, Bangladesh; 5 Department of Parasitology, School of Medicine, Infectious Diseases Research Center, Gonabad University of Medical Sciences, Gonabad, Iran; 6 Department of Pathobiology, Faculty of Veterinary Medicine, University of Tabriz, Tabriz, Iran; 7 Department of Medical Technology, School of Allied Health Sciences, Walailak University, Nakhon Si Thammarat, Thailand; 8 Center of Excellence Research for Melioidosis and Microorganisms (CERMM), Walailak University, Nakhon Si Thammarat, Thailand; 9 Akkhraratchakumari Veterinary College, Walailak University, Nakhon Si Thammarat, Thailand; 10 School of Pharmacy and Pharmacology, University of Tasmania, Hobart, TAS, Australia; 11 CICECO-Aveiro Institute of Materials, University of Aveiro, Aveiro, Portugal; 12 Department of Medical Sciences, University of Aveiro, Aveiro, Portugal; 13 Department of Biotechnology, AVIT, Vinayaka Mission’s Research Foundation (DU), Chennai Campus, Chennai, Tamil Nadu, India; 14 Department of Pharmaceutical Biology, Faculty of Pharmaceutical Sciences, UCSI University, Kuala Lumpur, Malaysia; 15 Institute for Tropical Biology & Conservation, University Malaysia Sabah, Malaysia; 16 Center for Health Sciences, Research Institute for Science and Technology, Polytechnic University of the Philippines, Sta. Mesa, Manila, Philippines; 17 Department of Biology, College of Science, Polytechnic University of the Philippines, Sta. Mesa, Manila, Philippines; 18 General Education Department, School of Languages and General Education, Walailak University, Nakhon Si Thammarat, Thailand; 19 Futuristic Science Research Center, School of Science, Walailak University, Nakhon Si Thammarat, Thailand

**Keywords:** anti-malarial candidates, galangin, lactate dehydrogenase, molecular docking, molecular dynamics, *Plasmodium falciparum*, Propolis extract, tectochrysin

## Abstract

**Background and Aim:**

Malaria continues to pose a global health challenge, exacerbated by the emergence of drug-resistant strains of *Plasmodium falciparum*. This study aimed to evaluate the anti-*Plasmodium* potential of Propolis extracts collected from various Iranian regions and to characterize the molecular interactions of their bioactive phytochemicals with *P. falciparum* lactate dehydrogenase (PfLDH), a key enzyme in parasite glycolysis.

**Materials and Methods:**

The anti-*Plasmodium* activity of ethanol-extracted Propolis was assessed against *P. falciparum* NF54 using the SYBR Green I fluorescence assay. Gas chromatography-mass spectrometry (GC-MS) analysis identified major phytochemicals in the most active extract. Molecular docking and 100-ns molecular dynamic (MD) simulations were performed to evaluate the binding affinity and stability of selected compounds (tectochrysin and galangin) against PfLDH in both holo (Protein Data Bank [PDB] ID: 1LDG) and apo (PDB ID: 2X8L) forms.

**Results:**

Propolis collected from Kermanshah city exhibited the highest anti-*Plasmodium* activity (IC_50_ = 6.69 ± 1.44 μg/mL). GC-MS analysis identified tectochrysin and galangin as major constituents. Molecular docking revealed strong binding affinities of tectochrysin (−7.8 kcal/mol) and galangin (−7.5 kcal/mol) to PfLDH, surpassing the binding energies of standard antimalarial drugs (chloroquine and quinine). MD simulations confirmed the stability of tectochrysin and galangin within the PfLDH active sites, with favorable root mean square deviation, root mean square fluctuation, gyration, solvent-accessible surface area, molecular surface area, and polar surface area profiles, indicating persistent and stable protein-ligand interactions throughout the simulation.

**Conclusion:**

The findings support the promising anti-*Plasmodium* potential of Propolis-derived compounds, particularly tectochrysin and galangin, as novel PfLDH inhibitors. Their potential applicability in transdisciplinary anti-parasitic therapy across human and veterinary medicine warrants further *in vivo* validation and clinical investigations.

## INTRODUCTION

Malaria, caused by *Plasmodium* spp., remains a major tropical disease affecting both human and animal populations. Although primarily considered a human disease, malaria also presents significant concerns in veterinary medicine due to its role in zoonotic transmission and the broader context of vector-borne diseases under the One Health framework. Notably, *Plasmodium knowlesi* has emerged as a formidable zoonotic pathogen, exemplifying the intricate connections among human, animal, and environmental health sectors [1]. In human populations, malaria continues to represent one of the leading causes of mortality and morbidity in endemic regions, particularly within low- and middle-income countries [2]. Severe complications arising from malaria infection include hypoglycemia, acute renal failure, cerebral malaria, and profound anemia [3]. Five species of *Plasmodium* – *Plasmodium falciparum*, *Plasmodium ovale*, *Plasm-odium malariae*, *Plasmodium knowlesi*, and *Plasmodium vivax* are recognized as causative agents of human disease. Among them, *P. vivax* is the most geographically widespread, whereas *P. falciparum* accounts for the highest number of fatalities [4]. According to the World Health Organization (WHO), approximately 627,000 malaria-related deaths occurred among 241 million reported cases across 85 countries in 2021.

Malaria is treatable using various WHO-approved anti-*Plasmodium* drugs, which are categorized into four primary classes: Antifolates, quinoline-related compounds, artemisinin (ART) derivatives, and antim-icrobials. However, the emergence of resistance to these established therapeutics has become a major global concern. In response to the critical gap created by escalating drug resistance [5], this study focuses on the evaluation of natural Propolis-derived phytochemicals as novel inhibitors of *P. falciparum*, offering a promising alternative pathway distinct from traditional synthetic antimalarial agents. Within the veterinary domain, hemoparasitic infections in domestic and wild animals further underscore the urgent need for broad-spectrum antiparasitic agents to mitigate disease transmission at the animal-human interface [6]. Given the rising resistance to conventional treatments, the exploration of bioactive natural compounds with cross-species efficacy, such as those derived from Propolis, holds significant promise for the development of innovative therapeutic strategies applicable to both human and veterinary medicine.

This situation highlights the pressing need to develop novel anti-*Plasmodium* agents. Natural products represent a rich and promising reservoir for alternative approaches to anti-malarial drug discovery [7]. Several plant extracts have demonstrated significant activity against *P. falciparum*, including those derived from *Polyalthia longifolia* [8], *Glycine max* seed extract [9], *Meriandra dianthera* leaf extract [10], and *Combretum racemosum* leaf extract [11]. Among natural sources, Propolis – a complex bee-derived product comprising plant resins, waxes, and secretions – has garnered attention for its broad biological activities, with its chemical composition varying according to botanical origin [12]. Although prior investigations have assessed the general anti-parasitic activity of Propolis, the present study uniquely identifies specific bioactive constituents, notably tectochrysin and galangin, that exhibit superior binding affinities and enhanced structural stability against PfLDH relative to standard antimalarial drugs.

Previous studies have reported the antip-arasitic potential of Propolis and its constituents against protozoan pathogens. Propolis samples collected from various regions in Iran demonstrated *in vitro* anti-*Plasmodium* activity, with half-maximal inhibitory concentration (IC_50_) values ranging from 16.2 to 80.0 μg/mL [13]. Similarly, Saudi Arabian Propolis extracts exhibited protective effects against *P. chabaudi*-infected mice, notably attenuating oxidative damage by reducing malondialdehyde levels while enhancing catalase (CAT) activity and gluta-thione levels [14]. *Plasmodium falciparum* lactate dehydrogenase (PfLDH) is a key enzyme that governs ATP production by catalyzing the conversion of lactate to pyruvate during the anerobic erythrocytic stages of the parasite’s life cycle [15]. Due to its essential role in parasite survival and its distinct amino acid composition relative to human lactate dehydrogenase [15], PfLDH has been proposed as an attractive molecular target for anti-*Plasmodium* drug development. Inhibiting PfLDH has been shown to compromise parasite viability, further validating its relevance as a therapeutic target [16].

Despite the availability of several WHO-approved antimalarial therapies, the rapid emergence of multidrug-resistant strains of *P. falciparum* presents a critical challenge to global malaria control efforts. Although synthetic compounds have been the cornerstone of malaria treatment, their efficacy is increasingly compromised, necessitating the search for novel therapeutic alternatives. Natural products, particularly those derived from Propolis, have demonstrated promising antiparasitic activities; however, specific bioactive compounds within Propolis remain underexplored for their direct inhibitory effects on validated molecular targets such as PfLDH. Prior studies have focused primarily on the crude anti*-Plasmodiu*m activity of Propolis extracts without detailed phytochemical profiling, structure-activity relationship elucidation, or mechanistic validation through molecular docking and dynamic simulation analyses. Furthermore, the potential of Propolis-derived compounds to serve as dual-purpose agents addressing both human and veterinary hemoparasitic infections has not been systematically investigated. Addressing these gaps is vital for advancing transdisciplinary, One Health-based antiparasitic strategies.

This study aimed to systematically investigate the antimalarial potential of Propolis extracts collected from diverse Iranian regions against *P. falciparum* NF54, with a particular focus on isolating and characterizing key bioactive constituents. Using a combination of *in vitro* drug susceptibility assays, gas chromatography–mass spectrometry (GC-MS) profiling, molecular docking, and molecular dynamics (MD) simulations, the study sought to evaluate the inhibitory efficacy and structural stability of candidate compounds targeting PfLDH. By identifying potent Propolis-derived inhibitors such as tectochrysin and galangin, this research aims to provide a scientific basis for the development of novel, naturally derived anti*-Plasmodiu*m therapeutics with potential cross-species applications in both human and veterinary medicine.

## MATERIALS AND METHODS

### Ethical approval

*In vitro* experiments with *P. falciparum* NF54 followed institutional biosafety and ethics guidelines. RBCs were obtained with informed consent from a healthy donor. Samples were anonymized. Ethical approval was granted by Mahidol University (Approval No. TMEC 22-057).

### Study period and location

Data collection for this study was conducted from April 2022 to May 2023 at the Laboratory of Pharmaceutical Biotechnology and Bioinformatics at Jashore University of Science and Technology in Jashore, Bangladesh; the Faculty of Tropical Medicine, Mahidol University, Bangkok, Thailand; and Research Institute for Health Sciences (RIHS) at Walailak University in Nakhon Si Thammarat, Thailand.

### Parasite preparation

*P. falciparum* NF54 cells were cultivated in human erythrocytes using RPMI 1640 medium (Gibco; Grand Island, NY, USA) supplemented with 25 mM HEPES, 0.225% NaHCO_3_, 0.1 mM hypoxanthine, 25 μg/mL gentamicin, and 5% AlbuMax II (Gibco; Auckland, New Zealand) as per standard protocol. Human red blood cells (RBCs) were obtained from a healthy adult volunteer with blood type O.

The culture was maintained under an atmosphere containing 5% oxygen, 5% carbon dioxide, and 90% nitrogen, as described by Trager and Jensen [17]. To synchronize the parasites to the ring stage, a 5% D-sorbitol solution (Avantor Performance Material LLC, Central Valley, USA) was used. The synchronized ring-stage parasites were consistently propagated in a suspension of RBCs with a parasitemia level of 1% and a hematocrit level of 2%. The cultures were incubated at 37°C.

### Preparation of Propolis extracts

Propolis extracts were sourced from various geographical regions across Iran to capture a broad spectrum of bioactive compounds. The geographical variability of sampling sites influences the chemical composition and diversity of the bioactive constituents present in Propolis. To obtain the extract, 20 g of Propolis was blended with 50 mL of 95% ethanol and left at room temperature (25°C) for a week. The substance was filtered using Whatman No. 1 filter paper (Whatman International Ltd., Maidstone, UK). The resulting alcoholic extract was evaporated in a vacuum before being dried using a rotary evaporator.

The dried extracts were stored at 4°C and subsequently dissolved in dimethyl sulfoxide (DMSO) at a concentration of 100 mg/mL for use.

Artesunate served as the positive control at concentrations of 0.78–100 ng/mL, alongside solvent-only negative controls, to ensure assay validity and benchmark for comparative efficacy assessment.

In this study, the Propolis extract was prepared using DMSO as the sole solvent. To ensure that the solvent did not interfere with the assay, the final DMSO concentration was diluted to below 0.5%, a level considered non-toxic to *P. falciparum* *in vitro*. The extract exhibited IC_50_ values ranging from 6.69 to 29.34 μg/mL.

### Drug susceptibility assay

The parasites in the ring stage were diluted to a parasitemia level of 1% in 2% hematocrit. Propolis extracts were prepared by diluting the drugs in 96-well plates using a two-fold serial dilution method. All tests included a positive control with artesunate concentrations ranging from 0.78 to 100 ng/mL. Propolis extract concentrations were systematically prepared in serial dilutions ranging from 0.98 ng/mL to 2,000 μg/mL, ensuring the inclusion of sufficient technical replicates (minimum of three) for each concentration to enhance reliability. The plates were then placed in a 37°C incubator for 72 h. After treat-ment with a drug, parasite growth was assessed using SYBR Green I, which was mixed with lysis buffer containing 20 mM Tris hydrochloride, 5 mM EDTA, 0.008% Saponin, and 0.08% Triton X [18, 19]. The reaction was incubated for 30 min in an unlit container at 25°C. The fluorescence intensity was measured at excitation and emission wavelengths of 485/520 nm. Parasite survival was determined by comparing a sample treated with a drug with that of a parasite growth control without any treatment. The resulting signal was then adjusted by normalizing it with the background signal obtained from the highest concentration of each drug. GraphPad Prism version 9.0.0 was used to plot the dose-response curves to determine the IC_50_. *In vitro* susceptibility data were expressed as IC_50_) values using GraphPad Prism version 9.0.0. IC_50_ values for each compound were reported as mean ± standard deviation, providing a clear representation of central tendency and variability. Paired t-tests were conducted to compare the IC_50_ values of each plant extract with those of the standard control, artesunate. Statistically significant differences were observed between all plant extracts and artesunate, with p < 0.05 considered indicative of statistical significance.

### GC-MS analysis

The extract used in this study was previously analyzed for its chemical composition in Sama-Ae *et al*. [20]. In brief, Propolis extract (20 mg/mL) was diluted with ethanol at a ratio of 1:10. The mixture was then centrifuged at 10,000 rpm for 10 min at a temperature of 10°C. GC-MS analysis was performed using an Agilent 7890A chromatograph coupled with a 5977A mass selective detector (Agilent, California, USA). The calibration procedures, internal standards used, and accuracy checks were detailed to confirm the method’s precision. The analysis was submitted to the Office of Scientific Instrument and Testing (OSIT) at Prince of Songkla University in Southern Thailand, which adheres to the international standard protocols and uses a validated database. Emphasis was placed on compounds with a match factor of ≥90% during the interpretation of the results. A VF-WAXms capillary column (30 m × 250 μm × 0.25 μm) film was used. Helium gas was used as the carrier at a flow rate of 1 mL/min. The column temperature was initially set at 60°C and then increased to 160°C at 10°C/min. The temperature was further increased to 325°C at 2.5°C/min. Finally, a 15-min hold time was observed at this temperature. The mass spectra were acquired in full-scan mode using a 70-eV ionization voltage, covering the m/z 35–500 range. The chemical ingredients were identified by comparing their mass spectrum data with data from the Wiley Library.

### *In silico* study

#### Compound selection

PfLDH is a potential drug target [21]. Compounds identified by GC-MS (tectochrysin, galangin, among others) were systematically selected based on their abundance and known bioactivity profiles. The compounds were selected according to their presence in the extract, followed by their relative abundance and established pharmacological significance. The analysis was submitted to the Office of Scientific Instruments and Testing (OSIT) at Prince of Songkla University in southern Thailand, which adheres to international standard protocols and uses a validated database. Emphasis was placed on compounds with a match factor of ≥90% during the interpretation of results. Their structural preparation involved optimization through the MMFF94 force field using Open Babel software to ensure structural accuracy before docking simulations [22]. The purpose of this study was to determine their ability to bind to the active site of PfLDH and effectively inhibit the enzyme. Chloroquine and quinine, which have proven to be effective against *P. falciparum*, were used as positive controls to increase the rigor of the computational antimalarial drug screening.

### Receptor preparation

Protein targets PfLDH (Protein Data Bank [PDB] IDs: 1LDG and 2X8L) were carefully prepared by removing crystallographic waters, inhibitors, and heteroatoms; adding polar hydrogens; and performing energy minimization to ensure structural integrity and consistency for docking. The protein was chosen in both states to account for structural variations between the ligand-bound and unbound states, which helps explain how chemicals interact with the enzyme in distinct conformations. PDB 1LDG corresponds to PfLDH with bound nicotinamide adenine dinucleotide (NADH) and oxamate, whereas PDB 2X8L represents the apoenzyme of PfLDH without NADH and oxamate. To prepare for docking, we removed water molecules from the crystallographic structure of the protein and eliminated any attached inhibitor molecules and other heteroatoms from 2X8L. This allowed the docking of ligands within the protein’s pockets. In addition, we included polar hydrogen atoms because crystallographic structures often lack hydrogen atoms. We used PyMOL software [23] to add polar hydrogen atoms and remove water molecules, heteroatoms, and inhibitor molecules. Finally, the protein molecules were saved in PDB format.

### Docking parameter setup

The grid box represents the entire receptor protein molecule. The parameters for each protein’s grid box are as follows:

- 1LDG: Center coordinates X: 23.91, Y: 16.96, Z: 30.13; Dimensions X: 88.68, Y: 71.09, Z: 100.01 (Å).
- 2X8L: Center coordinates X: 13.81, Y: 23.97, Z: 5.77; Dimensions X: 74.04, Y: 77.57, Z: 74.64 (Å).

### Ligand preparation

The ligand compounds were obtained from PubChem [24] in.sdf format. The molecules were optimized using the MMFF94 force field in Open Babel software and were saved in.pdb format.

### Molecular docking

Blind docking was used to screen potential inhibitory phytochemicals. In AutoDock Vina, a grid box encompassing the entire protein molecule was developed. Blind docking helped identify the regions where the ligand successfully interacted with the protein molecules. An exhaustiveness value of 16 was used to enhance the search thoroughness. AutoDock Vina [25] generated nine docked poses for each ligand compound, with Pose 1 showing the highest binding affinity. For further analysis, the Pose 1 structures were used. In the PyMOL study, both cartoon and 3D diagrams were generated. In addition, Discovery Studio Software (Dassault Systèmes SE, France) [26] was used to create 2D diagrams illustrating interactions between ligands and protein residues.

### MD simulations and trajectory analysis

An integrated approach was used, combining blind docking and extensive MD simulations, to robustly characterize ligand-protein stability and predict the functional inhibitory mechanisms of Propolis compounds against PfLDH. MD simulations were rigorously conducted using Schrödinger’s Desmond software (v3.6) (Schrödinger, Inc., NY, USA), clearly defining the simulation parameters including selection of the OPLS-2005 force field, temperature maintenance at 300 K, pressure control at 1 bar, solvation model (Transferable Intermolecular Potential with 3 Points [TIP3P]), and explicit ion inclusion (Na+, Cl-) at physiological concentrations (0.15 M). A Nosé–Hoover thermostat and isotropic Martyna–Tobias–Klein barostat were used to equilibrate the system under number of particles, pressure, and temperature (NPT) conditions, followed by 100-ps recording intervals with an energy of 1.2 kcal/mol. A 100-ns MD simulation was conducted to evaluate the structural stability of the target-ligand complexes. The OPLS-2005 force field and TIP3P water model within orthorhombic periodic boundary conditions were employed [27]. Sodium (Na+) and chloride (Cl-) ions were introduced to neutralize system charges at 0.15 M salt concentration. The NPT ensemble maintained a constant temperature (300 K) and pressure (1 atm). Simulations were conducted with 100-ps capturing intervals, achieving a recording efficiency of 1.2 kcal/mol. The Simulation Interaction Diagram (SID) tool of the Schrödinger package was used to evaluate the quality of the MD trajectory and analyze system behavior throughout the simulation. The stability of the protein-ligand complex was assessed using root mean square deviation (RMSD), root mean square fluctuation (RMSF), solvent-accessible surface area (SASA), radius of gyration (Rg), molecular surface area (MolSA), polar surface area (PSA), and hydrogen bonding interaction analysis [28].

## RESULTS AND DISCUSSION

### Emergence of drug-resistant malaria and the need for novel therapeutics

In 2021, malaria had a high mortality rate, affecting approximately 247 million people world- wide [29]. Although several antimalarial drugs, including chloroquine, chloroguanide (proguanil), sulfadoxine/pyrimethamine, quinine, mefloquine, halofantrine, ART, and atovaquone, have been deve-loped against *Plasmodium* parasites, the emergence of drugresistant strains poses a serious One Health concern. The development of drug resistance impacts not only human health but also veterinary health, especially in regions where *Plasmodium* spp. and close-ly related hemoparasites infect both humans and animals [30–33]. Therefore, a transdisciplinary ap- proach involving medical, veterinary, and environ-mental scientists is necessary to develop sustainable vector control strategies and novel therapeutic ag- ents for human and animal populations alike [34, 35].

### *In vitro* anti-*Plasmodium* activity of Propolis extracts

Propolis, a natural bee product composed of insect secretions, saliva, wax, and plant resins [36], demonstrated significant promise as an anti-*Plasmodium* agent in this study, with IC_50_ values ranging from 6.691 to 29.345 μg/mL. The anti-*Plasmodium* activity (IC_50_) of the Propolis extracts is presented in Table 1.

**Table 1:** The of IC_50_ Propolis extract on *Plasmodium falciparum* NF54.

Code	Compounds	Coordinate	Mean of IC_50_ (µg/mL) ± SD
IL 01	Kermanshah city Propolis	34.3277° N, 47.0778° E	6.69 ± 1.44
IL 03	Sarab city Propolis	37.9429° N, 47.5384° E	10.37 ± 1.64
IL 04	Tehran city Propolis	35.6892° N, 51.3890° E	15.51 ± 4.95
IL 06	Tabriz city Propolis	38.0962° N, 46.2738° E	8.81 ± 1.94
IL 08	Neyshabur city Propolis	36.2141° N, 58.7961° E	10.47 ± 0.12
IL 09	South Khorasan province Propolis	32.5176° N, 59.1042° E	29.34 ± 7.67
	Artesunate	-	0.002 ± 0.28

Data were presented as mean (μg/mL) from two biological duplicate experiments. SD=Standard deviation, IC_50_=Half-maximal inhibitory concentration

According to the WHO criteria, anti-*Plasmodium* activity was classified as very active at IC_50_ values <5 μg/mL, promising at 5–15 μg/mL, moderate at 15–50 μg/mL, and inactive at >50 μg/mL [37, 38]. Five Propolis extracts showed a promising level of activity, with IC_50_ values between 6.69 ± 1.44 and 15.51 ± 4.95 μg/mL. In addition, one extract demonstrated a moderate activity level, with an IC_50_ value of 29.34 ± 7.67 μg/mL. The Propolis sample collected from Kermanshah city showed good inhibitory activity compared to the positive control artesunate, with an IC_50_ value of 0.002 ± 0.28 μg/mL. Therefore, the Kermanshah city Propolis was selected for phytochemical profiling.

### GC-MS-based phytochemical profiling of Propolis

GC-MS analysis of the ethanol extract of Propolis identified 52 distinct chemical constituents. These peaks correspond to bioactive compounds, identified by comparing their peak area (%), retention time, height (%), and mass spectral fragmentation patterns with those of known compounds described in the Wiley Library. The primary chemical constituents (Table 2) were chrysin (18.86%), pinocembrin (15.02%), tectochrysin (9.88%), pinostrobin (4.93%), galangin (4.51%), 2,6-cresotaldehyde (3.62%), cinnamic acid (1.92%), β-selinenol (1.87%), cinnamylidene acetic acid (1.62%), proximadiol (1.62%), α-eudesmol (1.61%), heptacosane (1.48%), octacosanol (1.43%), trans-ferulic acid (1.19%), γ-eudesmol (1.17%), 3,4-dihydro-2-(1-naphthalenylmethylene)-1(2H)-naphthalenone (1.06%), and cis-vaccenic acid (1.01%).

**Table 2:** Gas chromatography–mass spectrometry analysis of ethanol extract of Kermanshah city Propolis (IL01).

RT	Compound	Formula	Match Factor	% of total
7.9433	Benzyl alcohol	C_7_H_8_O	97.1	0.33
9.5586	Benzeneethanol	C_8_H_10_O	98.5	0.81
10.6765	Benzoic acid	C_7_H_6_O_2_	87.4	0.11
11.4681	2,3-Dihydrobenzofuran	C_8_H_8_O	89.8	0.37
13.1904	Styryl carbinol	C_9_H_10_O	95.5	0.15
13.3188	*p*-vinylguaiacol	C_9_H_10_O_2_	95.6	0.51
14.0676	Benzenepropanoic acid, ethyl ester	C_11_H_14_O_2_	81.1	0.03
16.0519	2,6-Cresotaldehyde	C_8_H_8_O_2_	79.4	3.62
17.2501	2-isopropenyl-4A,8-dimethyl-1,2,3,4,4A,5,6,8A-octahydronaphthalene	C_15_H_24_	81.9	0.02
17.6512	D-gamma-cadinene	C_15_H_24_	88	0.08
17.8491	∆-Cadinene	C_15_H_24_	90.5	0.07
18.1486	α-Copaen-11-ol	C_15_H_24_O	94.7	0.23
18.5605	γ-benzylidene-butyric acid	C_11_H_12_O_2_	95.6	0.47
18.7049	trans-Nerolidol	C_15_H_26_O	91.9	0.25
20.1972	γ-Eudesmol	C_15_H_26_O	98.0	1.17
20.3737	tau. -Cadinol	C_15_H_26_O	90.9	0.2
20.5983	β-Selinenol	C_15_H_26_O	99.2	1.87
20.6625	α-Eudesmol	C_15_H_26_O	96.2	1.61
21.9569	Cinnamylidene acetic acid	C_11_H_10_O_2_	89.4	1.62
23.5562	1-(hydroxymethyl)-2,5,5,8A-tetramethyldecahydro-2-naphthalenol	C_15_H_28_O_2_	80.3	0.07
23.845	4,4-Dimethyladamantan-2-ol	C_12_H_20_O	84.0	0.33
23.952	Proximadiol	C_15_H_28_O_2_	97.9	1.62
25.1234	4,4,8-Trimethyltricyclo [6.3.1.0 (1,5)] dodecane-2,9-diol	C_15_H_26_O_2_	80.0	0.11
25.9417	o-Methylferulic acid	C_11_H_12_O_4_	91.8	0.23
26.9045	n-Hexadecanoic acid	C_16_H_32_O_2_	91.5	0.33
27.3377	cis-Z-. α.-Bisabolene epoxide	C_15_H_24_O	87.9	0.21
27.4928	Hexadecanoic acid, ethyl ester	C_18_H_36_O_2_	94.6	0.23
27.9635	Hexadecanal	C_16_H_32_O	85.1	0.12
28.7391	α-Linoleic acid	C_18_H_32_O_2_	90.9	0.13
29.4986	Nerolidyl acetate	C_17_H_28_O_4_	82.6	0.47
29.6751	*p*-Coumaric acid	C_9_H_8_O_3_	88.5	0.26
30.1779	cis-Vaccenic acid	C_18_H_34_O_2_	97.1	1.01
30.6325	Ethyl oleate	C_20_H_38_O_2_	93.5	0.94
31.1139	Octadecanoic acid, ethyl ester	C_20_H_40_O_2_	80.7	0.07
31.3386	trans-Ferulic acid	C_10_H_10_O_4_	80.9	1.19
32.2639	Cinnamic acid	C_10_H_10_O_4_	80.7	1.92
32.879	Heneicosane	C_21_H_44_	92.7	0.43
34.398	Pinostrobin	C_16_H_14_O_4_	99.3	4.93
34.612	Cinnamyl cinnamate	C_18_H_16_O_2_	90.1	0.55
35.8047	Pinocembrin	C_15_H_12_O_4_	98.4	15.02
36.0722	Pentacosane	C_25_H_52_	92.0	0.83
37.3826	Tectochrysin	C_16_H_12_O_4_	96.0	9.88
37.8907	3-Hydroxy-2-(4-hydroxy-3-methoxyphenyl)-4H-chromen-4-one	C_16_H_12_O_5_	85.0	0.66
38.8214	Chrysin	C_15_H_10_O_4_	93.6	18.86
39.0514	Heptacosane	C_27_H_56_	91.6	1.48
39.3509	3,4-Dihydro-2-(1-naphthalenylmethylene)-1 (2H)-naphthalenone	C_21_H_16_O	77.4	1.06
39.4632	Galangin	C_15_H_10_O_5_	87.0	4.51
44.0685	n-Tetracosanol-1	C_24_H_50_O	90.6	0.66
44.3841	Hentriacontane	C_31_H_64_	79.9	0.22
44.7906	Vitamin E	C_29_H_50_O_2_	70.6	0.06
46.5396	Octacosanol	C_28_H_58_O	93.7	1.43
47.8126	Handianol	C_30_H_50_O	75.7	0.12

RT=Retention time

Other studies from different countries have reported that Propolis contains various chemical compounds, including flavonoids, phenylpropanoids, terpenes, stilbenes, lignans, coumarins, and their pren-ylated derivatives. These compounds vary depending on geographical location, plant source, and bee species, resulting in different types of Propolis with distinct characteristics and properties [39, 40].

### *In silico* molecular docking analysis targeting PfLDH

Molecular docking studies predicted significant interactions between Propolis-derived compounds and PfLDH. This technique predicts the interactions between two molecules and a target protein [41]. In this study, we selected PfLDH as the target protein because it is essential for ATP generation in parasites. Moreover, PfLDH is present in all human-infecting *Plasmodium* species, including *P. falciparum*, *P. vivax*, *P. ovale*, *P. malariae*, and *P. knowlesi* [42].

Table 3 presents the predicted binding energies of 11 compounds identified in Propolis and control drugs with the PfLDH apoenzyme (PDB 2X8L) and holoenzyme (PDB 1LDG).

**Table 3:** Predicted binding energies of 11 ligands from Propolis to 1LDG and 2X8L.

No.	Compound name	Predicted binding Energy (∆G = −kcal/mol)	

		1LDG (holo)	2X8L (apo)
1	Delta-Cadinene	−7.2	−7.0
2	Alpha-Eudesmol	−7.1	−6.8
3	Gamma-Eudesmol	−6.5	−6.8
4	Beta-Selinenol	−6.2	−6.6
5	Trans-Ferulic acid	−5.9	−5.4
6	Trans-Nerolidol	−5.4	−4.9
7	Pentacosane	−4.2	−4.9
8	Chrysin	−7.5	−7.2
9	Galangin	−7.5	−7.0
10	Pinocembrin	−7.7	−7.1
11	Tectochrysin	−7.8	−7.1
12	Chloroquine (control)	−6.4	-
13	Quinine (control)	-	−7.0

The predicted binding energies of the ligands to both the apoenzyme and holoenzyme forms were similar. The four compounds with the lowest binding energies to the holoenzyme (1LDG) were tectochrysin, pinocembrin, galangin, and chrysin, with binding energies of −7.8, −7.7, −7.5, and −7.5 kcal/mol, respectively. The corresponding binding energies of these four compounds with the apoenzyme (2X8L) were −7.1, −7.1, −7.0, and −7.2 kcal/mol, respectively. Moreover, chloroquine was employed as a reference drug, demonstrating a binding affinity of −6.4 kcal/mol with the holoenzyme PfLDH (PDB-1LDG). In addition, quinine served as the control drug for the apoenzyme PfLDH (PDB-2X8L), exhibiting an excellent binding score of −7.0 kcal/mol. Thus, these four compounds showed potential for inhibiting the lactate dehydrogenase enzyme from *P. falciparum* and may serve as potential preventives or therapeutics against malaria.

### Selection and analysis of lead compounds: Galangin and tectochrysin

Galangin and tectochrysin were selected for further study. The compounds exhibited good binding energies to PfLDH compared with the control drugs. In molecular docking analyses, more negative binding energy values indicate stronger ligand-protein interactions [16]. Galangin and tectochrysin showed strong interactions with the holoenzyme PfLDH (1LDG), with predicted binding energies of −7.5 and −7.8 kcal/mol, respectively, which were supe-rior to that of the control compound chloroquine (−6.4 kcal/mol). The apoenzyme PfLDH (2X8L) also showed strong interactions with the two selected molecules, galangin and tectochrysin, with binding energies of −7.0 and −7.1 kcal/mol, respectively, like the control drug quinine (−7.0 kcal/mol). Unlike earlier reports, our study demonstrates through comparative docking and dynamic simulations that tectochrysin and galangin exhibit higher binding affinity and better protein stability compared to chloroquine and quinine, suggesting superior therapeutic potential [43].

Tectochrysin displayed different binding patterns compared to galangin, forming a higher number of hydrogen bonds, electrostatic bonds, and hydrophobic bonds with the active site amino acid residues of both holo and apo PfLDH, which may explain its higher activity. Table 4 presents the amino acid residues in 1LDG and 2X8L that form electrostatic, hydrogen, and hydrophobic bonds with tectochrysin and galangin. Galangin showed strong interaction with amino acids Gly99, Ala98, Asp53, and Ile54 of the holoenzyme PfLDH (1LDG), forming multiple hydrogen, electrostatic, and hydrophobic bonds, suggesting its potential as an anti-malaria drug candidate. According to the previous report by Tasdemir *et al*. [44], galangin exhibits anti-*Plasmodium* activity against the *P. falciparum* NF54 strain, with an IC_50_ of 39.4 μM and 0.002 μg/mL, respe-ctively. In conclusion, tectochrysin and galangin can be effective anti-*Plasmodium* therapeutic agents in the future.

**Table 4:** Interacting amino acid residues in 1LDG and 2X8L forming electrostatic, hydrogen, and hydrophobic binds with tectochrysin and galangin

Interaction	Name	Distance	Category	Types
Tectochrysin and 1LDG	THR97	2.36	Hydrogen Bond	Conventional Hydrogen Bond
	ASP53	3.85	Electrostatic	Pi-Anion
	ASP53	3.52	Electrostatic	Pi-Anion
	GLY99	3.23	Hydrogen Bond	Pi-Donor Hydrogen Bond
Galangin and 1LDG	GLY99	2.36	Hydrogen Bond	Conventional Hydrogen Bond
	ALA98	3.36	Hydrogen Bond	Carbon Hydrogen Bond
	ASP53	3.69	Electrostatic	Pi-Anion
	ILE54	3.96	Hydrophobic	Pi-Sigma
	ALA98	3.48	Hydrophobic	Pi-Sigma
Tectochrysin and 2X8L	GLY29	2.34	Hydrogen Bond	Conventional Hydrogen Bond
	GLY99	2.86	Hydrogen Bond	Conventional Hydrogen Bond
	ASP53	3.82	Electrostatic	Pi-Anion
	ASP53	3.37	Electrostatic	Pi-Anion
	ILE54	3.94	Hydrophobic	Pi-Sigma
	ILE54	3.75	Hydrophobic	Pi-Sigma
	ILE119	3.95	Hydrophobic	Pi-Sigma
Galangin and 2X8L	TYR174	4.07	Hydrophobic	Pi-Pi Stacked

### Visualization of ligand–PfLDH interactions

**Figure 1:**
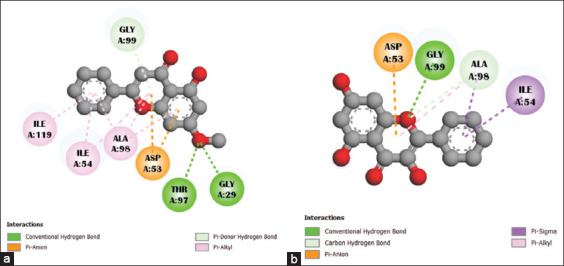
The interaction between selected ligand compounds (a) tectochrysin and (b) galangin with the targeted enzyme *Pf*LDH (Protein Data Bank: 1LDG) is represented. *Pf*LDH=*Plasmodium falciparum* lactate dehydrogenase.

**Figure 2:**
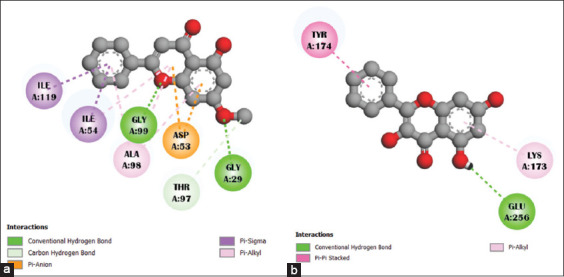
The interaction between selected ligand compounds (a) tectochrysin and (b) galangin with the targeted apoenzyme of *Pf*LDH (Protein Data Bank: 2X8L) is represented. *Pf*LDH=*Plasmodium falciparum* lactate dehydrogenase.

The 2D interactions of the compounds with PDB 1LDG (holoenzyme) and 2X8L (apoenzyme) are shown in Figures 1 and 2.

**Figure 3:**
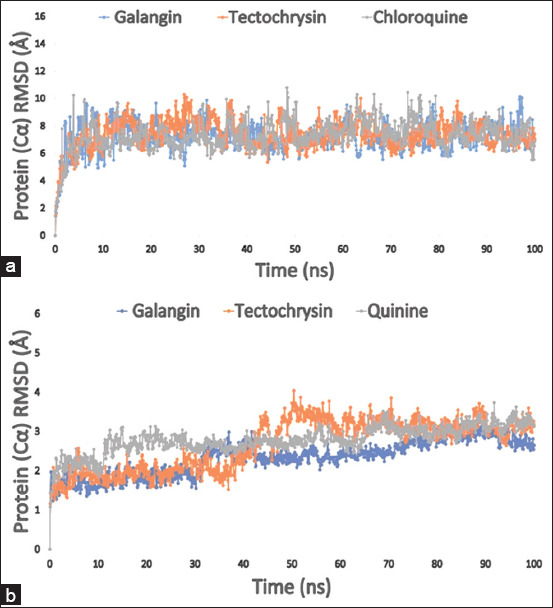
The RMSD value of the targeted enzyme *Pf*LDH (PDB: 1LDG) in complex with the ligands was calculated from the 100 ns simulation. (a) The RMSD value of the selected three compounds galangin, tectochrysin, and control chloroquine is represented by a blue, orange, and gray color, respectively; and (b) the RMSD value of the three compounds galangin, tectochrysin, and control quinine in association with the apoenzyme of *Pf*LDH (PDB: 2X8L) is shown. The colors blue, orange, and gray, respectively, reflect the three compounds’ RMSD values. PDB=Protein Data Bank, RMSD=Root mean square deviation, *Pf*LDH=*Plasmodium falciparum* lactate dehydrogenase.

The interactions of tectochrysin and galangin with the amino acid residues of 1LDG and 2X8L are presented in Table 4. The essential amino acid residues interacting with the ligands are indicated in bold (Figure 3). The results showed that both tectochrysin and galangin interact with the active site amino acids in PfLDH (either apo or holo forms) and can inhibit *P. falciparum*, as demonstrated in Figures 1 and 2 and Table 4.

### Protein stability and flexibility assessment via MD simulation

A compound with ligands can be used to assess a protein’s stability, flexibility, and intermolecular interactions through MD modeling [45–47]. This type of modeling is commonly used in computer-aided drug discovery. In our study, we conducted a 100-ns MD simulation to examine conformational changes in proteins caused by specific ligands. The stability of each protein-ligand complex was assessed by comparison with the simulation results of a known reference inhibitor [48]. The MD simulation findings were then analyzed using RMSD, RMSF, Rg, SASA, MolSA, PSA, and the intramolecular hydrogen bond between the protein and ligand [49].

The RMSD values of the complex systems were used to determine the stability of the compounds. In contrast, the RMSF values provide insight into the compactness of the protein-ligand complexes by measuring their average fluctuation [50]. By calculating the RMSD of the protein-ligand complexes using Cα atoms, we observed low protein fluctuation. Similarly, the RMSF values indicated minimal fluctuations in the complex system, suggesting that the ligands remained stably bound to the target protein. Other factors such as Rg, number of hydrogen bonds, SASA, MolSA, and PSA were also considered to further assess the stability of the complexes. This study conducted a 100-ns MD simulation using the Schrödinger package software (Desmond Application). The simulation was performed using relevant physiological and physicochemical parameters [48]. Except for slight variations, galangin and tectochrysin compounds showed similar RMSD and RMSF values when complexed with the holoenzyme PfLDH and the apoenzyme PfLDH compared with the control substances. The simulation findings for all other parameters were positive, suggesting that these compounds can be developed into anti-malarial medications.

### Dynamic stability of protein–ligand complexes

During the 100-ns MD simulation, the “Simulation Interaction Diagram (SID)” was used to explore the intermolecular interactions between proteins in complex with specific ligands [51, 52]. Throughout the simulation, the compounds formed multiple interactions, including hydrogen bonds, hydrophobic interactions, ionic bonds, and water-bridge bonds, which were maintained until the end of the simulation. This facilitated the formation of stable binding between the two target proteins.

Changes in the PfLDH protein due to mutations could affect ligand-protein interactions in the studied conserved interacting regions [53]. Genomic polymorphisms may alter a protein’s three-dimensional structure and affect ligand binding. These alterations can also affect the functional properties of proteins [54]. Investigating the specific effects of these polymorphisms on the functional elements of PfLDH could be beneficial for future research.

### RMSD and RMSF analyses

The compounds’ relative molecular size (RMSD) with respect to the enzymes was determined using C atoms. The RMSD is commonly used to measure the dissimilarity between observed and estimated values. If the results fall outside the allowable range, substantial alterations in the protein structure are observed. The average RMSD value is expected to vary between 1 and 3 Å (0.1–0.3 nm) from one frame to another.

**Figure 4:**
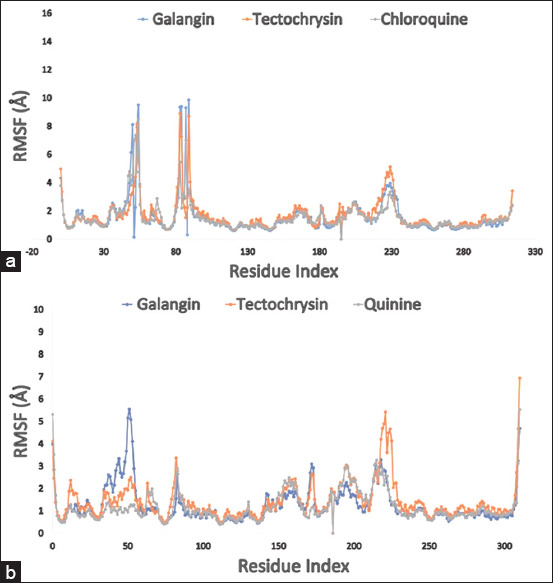
The graph displays the complex structure’s RMSF values, which were taken from the carbon atoms of protein residues. (a) The selected three compounds galangin, tectochrysin, and control chloroquine in complex with the targeted enzyme *Pf*LDH (PDB: 1LDG) are represented by a blue, orange, and gray color, respectively; (b) the colors blue, orange, and gray, respectively, indicate the RMSF values of the three substances galangin, tectochrysin, and control quinine in association with the apoenzyme of *Pf*LDH (PDB: 2X8L). PDB=Protein Data Bank, *Pf*LDH=*Plasmodium falciparum* lactate dehydrogenase, RMSF=Root mean square fluctuation.

Figure 4a compares the RMSD values of two selected compounds, galangin (blue) and tectochrysin (orange), and the control chloroquine (gray), in complex with PfLDH (PDB 1LDG) to observe structural changes. Although the RMSD values were slightly elevated initi-ally, all compounds stabilized within the acceptable fluctuation range. The average RMSD values for the apoenzyme PfLDH (PDB 2X8L) complexes with galangin (blue), tectochrysin (orange), and control quinine (gray) ranged from 1.5 to 3.5 Å (Figures 4a and b). Tectochrysin exhibited minor fluctuations between 35 and 65 ns of simulation time compared to the control drug quinine but gradually stabilized, displaying good stability for the remainder of the simulation.

RMSF can detect and quantify local changes in protein chains when specific residues interact with ligands. Figure 3a presents the computed RMSF values for the galangin (blue) and tectochrysin (orange) complexes with the holoenzyme PfLDH. In contrast, Figure 3b illustrates the RMSF values of the PfLDH enzyme in its unbound state. Changes in protein flexib-ility can be observed as certain chemicals bind to specific locations. Chloroquine and quinine were used as control drugs for comparison with the targeted compounds in terms of structural flexibility.

By analyzing the RMSF graph of the holoenzyme PfLDH (PDB 1LDG)–ligand complexes, all compounds, including the control, showed fluctuations between residues 50–54 and 83–89, with maximum fluctuation ranges reaching approximately 9.87 Å that gradually stabilized. Galangin (blue) and tectochrysin (orange) exhibited lower fluctuations than the control comp-ounds, indicating a more stable system.

Further analysis of the RMSF graph revealed extreme fluctuations between residues 38–54 for galangin and 217–225 for tectochrysin, suggesting slightly less stability for the compounds in the apoen-zyme PfLDH but still within an acceptable range. N- and C-terminal domains often exhibit the greatest diversity in proteins. Therefore, it is reasonable to infer that the investigated ligand compounds have a low probability of causing significant fluctuations in protein displacement under physiological conditions.

### Radius of Rg analysis

**Figure 5:**
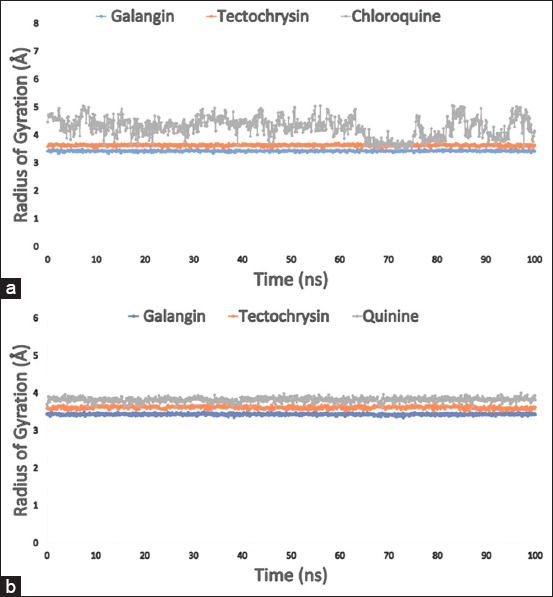
(a) The Rg value of 100 ns Molecular Dynamic Simulation (MDS) assessment for the targeted enzyme *Pf*LDH (PDB: 1LDG) in complex with the selected compounds galangin, tectochrysin, and control chloroquine is represen-ted by a blue, orange, and gray color, respectively; and (b) blue, orange, and gray, respectively, reflect the Rg values of the three substances galangin, tectochrysin, and control quinine in association with the apoenzyme of *Pf*LDH (PDB: 2X8L). PDB=Protein Data Bank, *Pf*LDH=*Plasmodium falciparum* lactate dehydrogenase, Rg=Radius of gyration.

The macromolecule must maintain a compact and rigid structure for stability during MD simulations. The statistical metric Rg, derived from MD simulation trajectories, quantifies the degree of compactness and rigidity of the molecules in their dynamic states. Furthermore, the protein undergoes folding and unfolding when the Rg value is low and high, respectively. The average Rg values for galangin, tectochrysin, and the control drug chloroquine bound to holoenzyme PfLDH (PDB 1LDG) were calculated as 3.4 Å, 3.5 Å, and 4.4 Å, respectively. This implies that the binding sites remained structurally intact after ligand binding (Figures 5a and b).

The stability of galangin, tectochrysin, and the control quinine bound to the apoenzyme PfLDH was also assessed. The values obtained from the simulation were 3.4 Å, 3.6 Å, and 3.8 Å, respectively. This result demonstrates that the protein’s binding site did not undergo significant structural changes upon binding to the selected ligands. In all complexes, the proximity between the highest and lowest Rg values supports the low deviation observed in the system.

### SASA, MolSA, and PSA analysis

**Figure 6:**
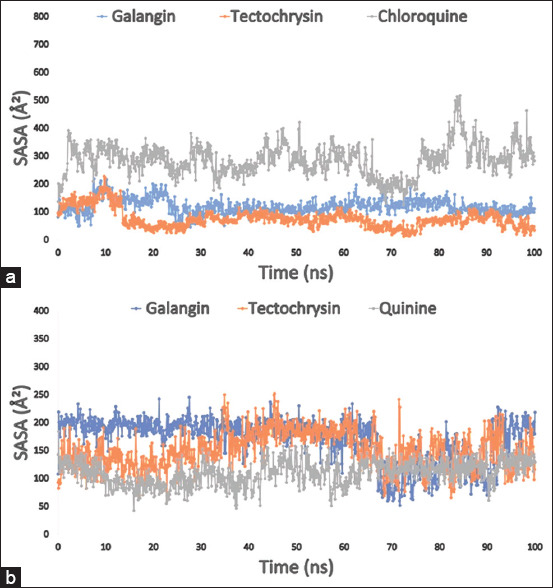
The SASA of the protein-ligand interaction complexes was calculated using the 100 ns simulated interaction diagram, (a) where the colors blue, orange, and gray represented the three selected ligands compound galangin, tectochrysin, and chloroquine in interaction with the targeted enzyme *Pf*LDH (PDB: 1LDG), respectively; and (b) the SASA value of the selected three compounds galangin, tectochrysin, and control quinine in complex with the apoenzyme of *Pf*LDH (PDB: 2X8L) represented by a blue, orange, and gray color, respectively. PDB=Protein Data Bank, *Pf*LDH=*Plasmodium falciparum* lactate dehydrogenase, SASA=Solvent-accessible surface area.

The SASA affects the structure and function of biological macromolecules. The amino acid residues located on the protein surface typically act as active sites and interact with other molecules and ligands. This helps to understand the solvent-like characteristics (hydrophilic or hydrophobic) of a molecule and protein-ligand complexes. Consequently, the SASA value of the protein, when combined with galangin, tectochrysin, and the control drugs chloroquine and quinine, was calculated, as shown in Figures 6a and b. The SASA values for the complex system ranged from 50 to 350 Å^2^ for the PfLDH enzyme and from 80 to 200 Å^2^ for the PfLDH apoenzyme. This indicates that a considerable portion of the amino acid residues was exposed to the selected substances in the complex system.

**Figure 7:**
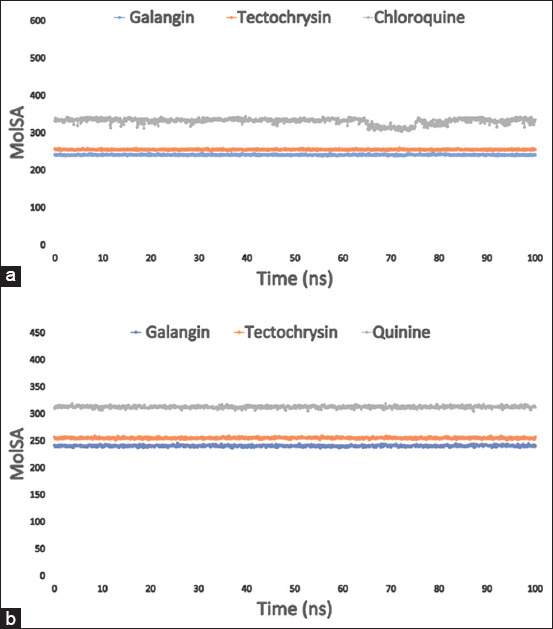
The radius of MolSA value of 100 ns MDS evaluations for the targeted enzyme *Pf*LDH (PDB: 1LDG) in complex with the three ligand compounds have been represented in the graphs, (a) where the selected three ligand compounds galangin, tectochrysin, and control chloroquine in complex with the protein are represented by blue, orange, and gray color, respectively; and (b) the colors blue, orange, and gray, respectively, show the MolSA values of the three substances galangin, tectochrysin, and control quinine in association with the apoenzyme of *Pf*LDH (PDB: 2X8L). PDB=Protein Data Bank, *Pf*LDH=*Plasmodium falciparum* lactate dehydrogenase, MolSA=Molecular surface area.

When the probe radius is set to 1.4, the MolSA equals the van der Waals surface area. Our computational work focused on studying the interactions between the ligand compounds galangin, tectochrysin, and the control chloroquine with the PfLDH enzyme. We also investigated the interactions of galangin, tectochrysin, and control quinine with the apoenzyme of PfLDH. The results revealed that all these compounds exhibited the typical van der Waals surface area, as shown in Figures 7a and b.

**Figure 8:**
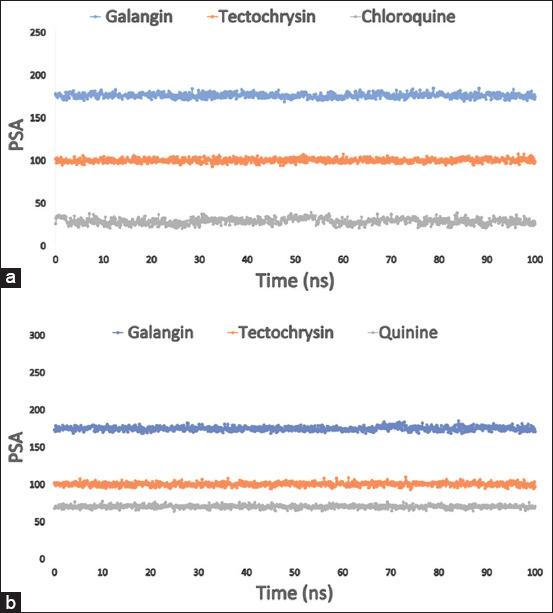
From the 100 ns simulated interaction diagram, (a) the PSA of the protein-ligand interaction compounds was estimated, where blue, orange, and grey colors represented the selected three ligands compound galangin, tectochrysin, and control chloroquine in contact with the enzyme *Pf*LDH (PDB: 1LDG), respectively; and (b) the PSA value of the selected three compounds galangin, tectochrysin, and control quinine in complex with the apoenzyme of *Pf*LDH (PDB: 2X8L) represented by a blue, orange, and gray color, respectively. PDB=Protein Data Bank, *Pf*LDH=*Plasmodium falciparum* lactate dehydrogenase, PSA=Polar surface area.

In addition, a structure’s PSA is determined solely by the oxygen and nitrogen atoms. In this study, the protein of interest exhibited a significant PSA value when interacting with galangin and tectochrysin ligand molecules in the presence of the PfLDH enzyme and PfLDH apoenzyme, as shown in Figures 8a and b.

### SID and hydrogen bond analysis

**Figure 9:**
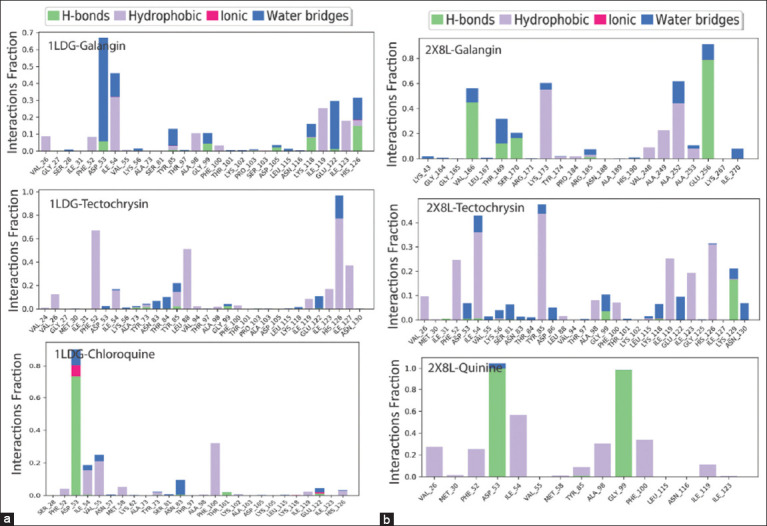
Ligand-protein interaction of compounds and controls. Interaction of galangin, tectochrysin, and chloroquine to 100 ns MD simulation with the enzyme *Pf*LDH (PDB: 1LDG) (a). Interaction of galangin, tectochrysin, and control quinine to 100 ns MD simulation with apoenzyme of *Pf*LDH (PDB: 2X8L) (b). PDB=Protein Data Bank, *Pf*LDH=*Plasmodium falciparum* lactate dehydrogenase, MD=Molecular dynamics.

The interactions between ligands and proteins, particularly hydrogen bonds, hydrophobic bonds, and water bridges, significantly affect drug selectivity, metabolism, and adsorption. Therefore, we determined the intermolecular interaction of the protein-ligand complex during the 100-ns simulation using the “Simulation Interaction Diagram (SID).” The interaction fraction value between the protein PfLDH and its ligands galangin, tectochrysin, and the control drug chloroquine was calculated and is shown in Figure 9a. In addition, Figure 9b illustrates the interaction of the ligands galangin, tectochrysin, and control quinine with the protein apoenzyme of PfLDH. The number of hydrogen bonds in a system enhances a potential drug’s adsorption and metabolic properties. Robust hydrogen interactions with protein residues were observed in all naturally occurring bioactive compounds.

### Broader implications and limitations

Our study revealed that Propolis extract inhibits *P. falciparum* NF54. *In silico* molecular docking analysis revealed that galangin and tectochrysin have the highest binding affinity. This study further suggests that galangin and tectochrysin inhibit Ca atoms in the protein-ligand complexes associated with malaria. Having tectochrysin and galangin exhibiting good binding affinities for PfLDH thereby provides credence for their possible uses as anti-malarial candidates. Notably, similar glycolytic enzymes are also involved in the metabolism of hemoparasites that affect livestock and companion animals (e.g., *Babesia* and *Theileria*). Original findings from this study indicate that tectochrysin and galangin, through their shared glycolytic target PfLDH, may exhibit broader-spectrum activity against other hemoparasitic organisms, thus uniquely contributing to the emerging field of transdisciplinary antiparasitic therapy [55].

However, this study had limitations in investigating phytochemicals and conducting molecular docking analysis on other bioactive extracts of Propolis against malaria from various regions of Iran. *In vivo* studies and clinical trials are highly recommended to validate the future anti-*Plasmodium* effects of galangin and tectochrysin. More comprehensive studies are also recommended on detecting pure compounds from naturally medicinal plants tested against this deadly pathogenic parasite that causes malaria.

## CONCLUSION

This study demonstrated that Iranian Propolis extracts exhibit significant inhibitory activity against *P. falciparum* NF54, with five extracts classified as promising according to the WHO criteria and IC_50_ values ranging from 6.69 to 15.51 μg/mL. Phytochemical analysis through GC-MS identified 52 bioactive consti-tuents, among which galangin and tectochrysin were selected for further *in silico* evaluation based on their abundance and pharmacological relevance. Molecular docking and MD simulations confirmed that galangin and tectochrysin exhibit stronger binding affinities and higher complex stability toward PfLDH compared to standard antimalarial drugs chloroquine and quinine. Furthermore, these compounds maintained stable protein-ligand interactions throughout 100-ns MD simulations and showed favorable structural and surf-ace properties (RMSD, RMSF, Rg, SASA, MolSA, and PSA analyses), highlighting their potential as promising antimalarial agents.

The primary strength of this study lies in its integrative approach, combining *in vitro* susceptibility testing with comprehensive *in silico* modeling to predict compound efficacy and interaction mechanisms. Another notable strength is the identification of glyco-lytic pathway enzymes such as PfLDH as broader tran-sdisciplinary antiparasitic targets, supporting future applications in both human and veterinary parasitology.

However, the study was limited by its exclusive focus on specific Propolis extracts and the absence of *in vivo* validation or cytotoxicity profiling against mammalian cells. Therefore, further experimental stu-dies, including animal model testing, cytotoxicity assays, and clinical trials, are strongly recommended to validate the therapeutic potential of galangin and tectochrysin against malaria and possibly other hemoparasitic infections.

Future research should also explore the isolation and characterization of pure compounds from diverse Propolis sources worldwide, coupled with mechanistic studies targeting other essential *Plasmodium* enzymes to broaden the scope of antimalarial drug discovery based on natural products.

## AUTHORS’ CONTRIBUTION

DAK, MNH, and VN: Conceptualized and designed the study. DAK, MNH, RB, SS, YS, AAS, AH, KC, PW, AS, RN, IS, WM, AKP, MdLP, SSS, TM, CW, RVL, SC, and VN: Data acquisition. DAK, MNH, RB, SS, YS, AAS, AH, KC, PW, AS, RN, IS, WM, AKP, MdLP, SSS, TM, CW, RVL, SC, and VN: Literature review and data analysis and interpretation. DAK, MNH, RB, SS, YS, AAS, AH, KC, PW, AS, RN, and VN: Drafted the manuscript. IS, WM, AKP, MdLP, SSS, TM, CW, RVL, SC, and VN: Revised the manuscript. All authors have read, reviewed, and approved the final manuscript.
